# Haemoglobin glycation index and risk for diabetes-related complications in the Action in Diabetes and Vascular Disease: Preterax and Diamicron Modified Release Controlled Evaluation (ADVANCE) trial

**DOI:** 10.1007/s00125-017-4539-1

**Published:** 2018-01-08

**Authors:** Sigrid C. van Steen, Mark Woodward, John Chalmers, Qiang Li, Michel Marre, Mark E. Cooper, Pavel Hamet, Giuseppe Mancia, Stephen Colagiuri, Bryan Williams, Diederick E. Grobbee, J. Hans DeVries, on behalf of the ADVANCE Collaborative Group

**Affiliations:** 1 Department of Endocrinology, Academic Medical Centre, University of Amsterdam, Postbus 22660, 1100 DD Amsterdam, the Netherlands; 2 The George Institute for Global Health, University of Sydney, Sydney, NSW Australia; 3 Department of Epidemiology, Johns Hopkins University, Baltimore, MD USA; 4 Department of Endocrinology, Hôpital Bichat-Claude Bernard, Université Paris, Paris, France; 5 Diabetes Domain, Baker IDI Heart and Diabetes Institute, Melbourne, VIC Australia; 6 Centre de Rechercher, Centre Hospitalier de l’Université de Montréal (CRCHUM), Montréal, Québec Canada; 7 Department of Medicine and Surgery, University of Milan-Bicocca, Milan, Italy; 8 Istituto Auxologico Italiano, Milan, Italy; 9 Boden Institute of Obesity, Nutrition and Exercise, University of Sydney, Sydney, NSW Australia; 10 National Institute of Health Research UCL Hospitals Biomedical Research Centre, London, UK; 11 Julius Clinical, Zeist, the Netherlands; 12 Julius Centre for Health Sciences and Primary Care, University Medical Centre Utrecht, Utrecht, the Netherlands

**Keywords:** (Blood) glucose, Cardiovascular complications, Diabetes mellitus, type 2, HbA_1c_, Hypoglycaemia, Mortality

## Abstract

**Aims/hypothesis:**

Previous studies have suggested that the haemoglobin glycation index (HGI) can be used as a predictor of diabetes-related complications in individuals with type 1 and type 2 diabetes. We investigated whether HGI was a predictor of adverse outcomes of intensive glucose lowering and of diabetes-related complications in general, using data from the Action in Diabetes and Vascular Disease: Preterax and Diamicron Modified Release Controlled Evaluation (ADVANCE) trial.

**Methods:**

We studied participants in the ADVANCE trial with data available for baseline HbA_1c_ and fasting plasma glucose (FPG) (*n* = 11,083). HGI is the difference between observed HbA_1c_ and HbA_1c_ predicted from a simple linear regression of HbA_1c_ on FPG. Using Cox regression, we investigated the association between HGI, both categorised and continuous, and adverse outcomes, considering treatment allocation (intensive or standard glucose control) and compared prediction of HGI and HbA_1c_.

**Results:**

Intensive glucose control lowered mortality risk in individuals with high HGI only (HR 0.74 [95% CI 0.61, 0.91]; *p* = 0.003), while there was no difference in the effect of intensive treatment on mortality in those with high HbA_1c_. Irrespective of treatment allocation, every SD increase in HGI was associated with a significant risk increase of 14–17% for macrovascular and microvascular disease and mortality. However, when adjusted for identical covariates, HbA_1c_ was a stronger predictor of these outcomes than HGI.

**Conclusions/interpretation:**

HGI predicts risk for complications in ADVANCE participants, irrespective of treatment allocation, but no better than HbA_1c_. Individuals with high HGI have a lower risk for mortality when on intensive treatment. Given the discordant results and uncertain relevance beyond HbA_1c_, clinical use of HGI in type 2 diabetes cannot currently be recommended.

**Electronic supplementary material:**

The online version of this article (10.1007/s00125-017-4539-1) contains peer-reviewed but unedited supplementary material, which is available to authorised users.


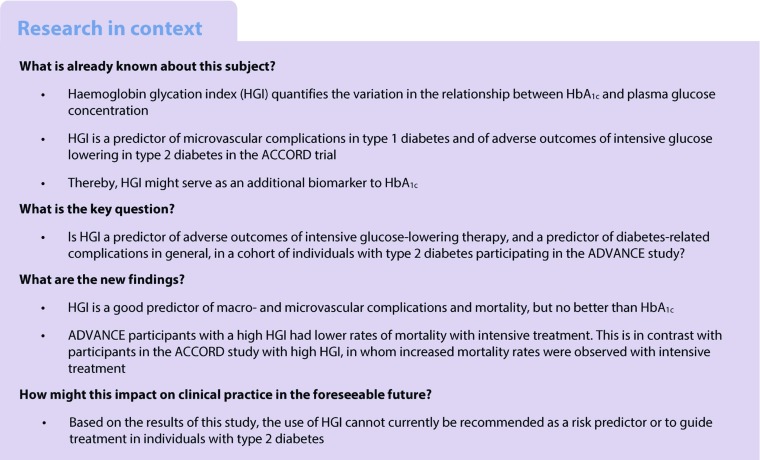


## Introduction

HbA_1c_ is an established means of monitoring average blood glucose levels and a surrogate marker of the effect of glucose-lowering interventions [1]. It is highly associated with the risk for diabetes-related complications, in particular those of microvascular origin [2–5]. Although HbA_1c_ is almost universally accepted to guide and monitor diabetes treatment, its use in clinical practice has arguable limitations. There is a proposed inter-individual variation in the propensity for glycation, in both healthy individuals and those with diabetes [6–13], limiting the use of HbA_1c_ as a one-size-fits-all measurement. Moreover, the value of HbA_1c_ as a surrogate endpoint was questioned by the results of the Action to Control Cardiovascular Risk in Diabetes (ACCORD) trial, where HbA_1c_ lowering may have had detrimental effects on the risk of premature mortality [14]. Therefore, ‘the lower the better’ may not universally hold for HbA_1c_, and additional (bio)markers might be useful to individualise treatment targets and risk prediction [15].

The haemoglobin glycation index (HGI) quantifies the variation in the relation between HbA_1c_ and the plasma glucose concentration [16]. For any individual within a study population, HGI is defined as the difference between the observed HbA_1c_ and the fitted value from a simple linear model that predicts HbA_1c_ from the fasting plasma glucose (FPG) concentration, i.e. the residual from the fitted linear regression line. In previous studies, HGI was normally distributed, stable over time and consistent over a wide range of blood glucose concentrations [17–20]. In an analysis in individuals with type 1 diabetes in the DCCT, a high HGI was associated with the risk for and progression of retino- and nephropathy [21]. In an analysis of the ACCORD trial, only participants in the highest HGI third were at higher risk for mortality and those with high HGI showed no benefit on cardiovascular outcomes after intensive glucose lowering, in contrast to participants with a low or intermediate HGI [16]. The use of HGI is not without controversy, as in the DCCT population it was shown that the effect of HGI on microvascular complications disappeared after adjustment for the effect of HbA_1c_ [22]. However, the use of HbA_1c_ in type 1 diabetes is undisputed, whereas in individuals with type 2 diabetes, HbA_1c_ seems to have shortcomings, as demonstrated by the ACCORD trial.

The aim of this study was to assess whether HGI is a predictor of adverse outcomes of intensive glucose-lowering therapy and a predictor of diabetes-related complications in the cohort of individuals with type 2 diabetes recruited for the Action in Diabetes and Vascular Disease: Preterax and Diamicron Modified Release Controlled Evaluation (ADVANCE) trial (ClinicalTrials.gov registration no. NCT00145925) [23]. Additionally, we aimed to compare the predictive values of HGI and HbA_1c_ to assess the possible added value of HGI beyond HbA_1c_.

## Methods

### ADVANCE trial

In the ADVANCE trial, 11,140 individuals with type 2 diabetes and a history of, or a risk factor for, vascular disease were randomised in a factorial design between two BP-lowering strategies and two glucose-lowering strategies [24]. Glucose-lowering treatment was either standard (based on local guidelines) or intensive, starting with gliclazide (30–120 mg daily, modified release) and adding other medication as necessary (based on the study protocol and discretion of the treating physician) to achieve a HbA_1c_ level of ≤48 mmol/mol (≤6.5%). Primary endpoints were a composite of major macro- and microvascular events. Study participants were, on average, 66 years old, with a mean diabetes duration of 8 years and a mean baseline HbA_1c_ of 59 mmol/mol (7.5%). After a follow-up of 5 years, mean HbA_1c_ was 48 mmol/mol (6.5%) in the intensively treated group vs 56 mmol/mol (7.3%) in the standard group. Intensive glucose-lowering treatment reduced the combination of macro- and microvascular events, mainly due to a 20% reduction in nephropathy, at the cost of an 86% increase in the risk of severe hypoglycaemia.

### Present study

#### HGI

**Fig. 1:**
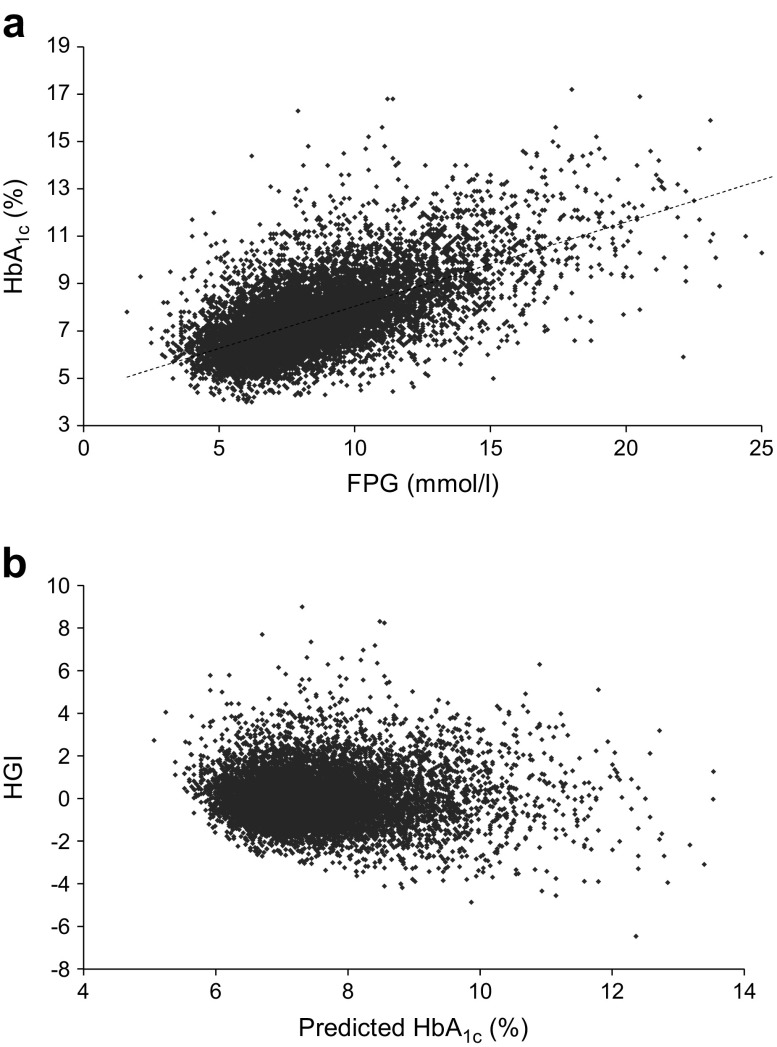
**(a)** Regression of FPG on HbA_1c_. The dotted line represents the simple linear regression line of the equation: HbA_1c_ (%) = 4.5 + 0.356 × FPG (mmol/l), *r*^2^ = 0.40. **(b)** Plot of the residuals (HGI) vs the fitted (predicted HbA_1c_) values (*p*=1.00). To convert values for HbA_1c_ in % into mmol/mol, subtract 2.15 and multiply by 10.929

We excluded 57 individuals with missing baseline HbA_1c_ (*n* = 54) or baseline FPG (*n* = 3). For the 11,083 remaining individuals, we fitted a linear regression. The linear regression equation describing the relation between baseline HbA_1c_ and FPG in our population was HbA_1c_ (%) = 4.5 + 0.356 × FPG (mmol/l) (*r*^2^ = 0.40, Fig. 1a). Results from simple linear regression were comparable with a cubic spline model, so we chose the simpler linear model. We derived predicted HbA_1c_ by inserting FPG values into this regression equation. Baseline HGI was calculated by subtracting predicted HbA_1c_ from observed HbA_1c_ (equalling the residual from the regression line, Fig. 1b). In this way, individuals with high HGI will have a higher measured HbA_1c_ than anticipated from the FPG value. In accordance with existing literature, we divided the population in three equally sized HGI groups (low, intermediate and high). This study was conducted according to the principles of the declaration of Helsinki [25] and in accordance with the Dutch Medical Research Involving Human Subjects Act (WMO). All participants provided written informed consent for the original study.

#### Outcomes

We analysed four predefined outcome measures: (1) major macrovascular events, defined as death from cardiovascular cause, non-fatal myocardial infarction or non-fatal stroke; (2) major microvascular events, defined as new or worsening nephropathy or retinopathy; (3) total mortality; and (4) severe hypoglycaemia. Severe hypoglycaemia was defined as transient dysfunction of the central nervous system that could not be self-treated.

#### Statistical analysis

We compared baseline characteristics between the low, intermediate and high HGI groups using ANOVA or Kruskal–Wallis tests for continuous variables, depending on the distribution, and *χ*^2^ tests for categorical variables. We used Cox proportional hazard regression models to analyse the association between baseline HGI and time to event for all four outcomes, with adjustment for covariates. We studied separately: (1) the treatment-modifying effect of HGI; and (2) the effect of HGI independent of treatment allocation—the first to make a comparison with the results from the ACCORD reanalysis, the latter as it seems more relevant for use in clinical practice where individuals will undergo variable treatment regimens. To assess whether HGI was better than time-matched (baseline) HbA_1c_ we added HbA_1c_ as a covariate to our models and used HbA_1c_ instead of HGI as predicting variable to compare the effect on outcomes.

#### Treatment-modifying effect of HGI

We investigated the effect of intensive glucose control on outcomes across HGI groups, by adding the interaction term between HGI group and treatment allocation (intensive or standard glucose-lowering therapy) to the model with HGI and treatment. We adjusted the model for age, sex, ethnic origin (Asian or non-Asian), BMI, duration of type 2 diabetes, history of macro- and microvascular events, current drinking and smoking habits, use of glucose-lowering medication, use of BP-lowering drugs, systolic and diastolic BP, haemoglobin, renal function (eGFR), LDL-, HDL- and total cholesterol and triacylglycerol. We added baseline HbA_1c_ to the before-mentioned covariates in a separate analysis. Finally, we assessed the associations between HbA_1c_ (instead of HGI) as the exposure variable and outcomes, adjusted for the same covariates and studied the interaction between treatment and HbA_1c_.

#### Effect of HGI independent of treatment allocation

We assessed the association between HGI and outcomes, irrespective of treatment, for the low, intermediate and high HGI group, as well as for HGI as a continuous variable. The low HGI group served as the reference group in the comparison. We adjusted for the identical set of standard covariates (with and without HbA_1c_). Again, we assessed the associations between HbA_1c_ (instead of HGI) as the exposure variable and outcomes, adjusted for the same covariates.

As only 1.5% of data were missing, no imputation was carried out. Significance levels were set at a *p* < 0.05. Statistical analyses were performed using SAS Enterprise Guide 7.1 (SAS STAT 9.4; SAS Institute, Cary, NC, USA).

## Results

In this population, HGI showed a normal distribution, ranging from −6.46 to 8.99, with a mean of 0 (SD 1.20). When dividing the study population into thirds, HGI cut-off points were ≤−0.53 for the low, −0.52 to 0.28 for the intermediate and ≥0.29 for the high HGI group. Mean baseline HGI was −1.14 (SD 0.57) in the low, −0.14 (SD 0.23) in the intermediate and 1.29 (SD 0.99) in the high HGI group (Table 1).

**Table 1:** Baseline characteristics of individuals by HGI group

Characteristic	Low HGI, *n*=3694	Intermediate HGI, *n*=3696	High HGI, *n*=3693	*p* value^a^
HGI (%)	−1.14 ± 0.57	−0.14 ± 0.23	1.29 ± 0.99	
HbA_1c_ (mmol/mol)	45.0 (40.0–52.0)	54.0 (49.0–60.0)	69.0 (61.0–81.0)	<0.0001
HbA_1c_ (%)	6.30 (5.80–6.90)	7.06 (6.60–7.60)	8.50 (7.71–9.60)	<0.0001
FPG (mmol/l)	8.10 (6.83–10.00)	7.60 (6.40–9.10)	8.10 (6.60–10.20)	<0.0001
Age (years)	65.89 ± 6.38	66.28 ± 6.43	65.18 ± 6.32	<0.0001
Female sex	1543 (41.8)	1524 (41.2)	1635 (44.3)	0.019
BMI (kg/m^2^)	28.36 ± 5.10	28.61 ± 5.26	28.05 ± 5.19	<0.0001
Diabetes duration (years)	6 (2–11)	6 (3–11)	8 (4–12)	<0.0001
Asian ethnicity	1234 (33.4)	1233 (33.4)	1750 (47.4)	<0.0001
Current smoker	499 (13.5)	578 (15.6)	598 (16.2)	0.003
Current drinker	1298 (35.1)	1245 (33.7)	835 (22.6)	<0.0001
Systolic BP (mmHg)	145.56 ± 21.56	144.81 ± 21.13	144.67 ± 21.90	0.164
Diastolic BP (mmHg)	80.87 ± 10.87	80.49 ± 10.93	80.54 ± 10.97	0.260
eGFR (ml min^−1^ [1.73 m]^−2^)	74.54 (62.53–87.60)	74.73 (62.25–88.17)	75.25 (61.37–89.88)	0.527
HDL-cholesterol (mmol/l)	1.28 ± 0.37	1.25 ± 0.34	1.24 ± 0.34	<0.0001
LDL-cholesterol (mmol/l)	3.11 ± 1.04	3.06 ± 1.00	3.16 ± 1.05	0.0001
Total cholesterol (mmol/l)	5.20 ± 1.19	5.14 ± 1.12	5.25 ± 1.26	0.001
Triacylglycerol (mmol/l)	1.60 (1.16–2.30)	1.60 (1.19–2.30)	1.70 (1.20–2.40)	0.001
Glucose-lowering medication	3256 (88.1)	3326 (90.0)	3496 (94.7)	<0.0001
Insulin	42 (1.1)	43 (1.2)	70 (1.9)	0.007
BP-lowering medication	2865 (77.6)	2773 (75.0)	2694 (72.9)	<0.0001
Past microvascular event	315 (8.5)	357 (9.7)	481 (13.0)	<0.0001
Past macrovascular event	1187 (32.1)	1179 (31.9)	1206 (32.7)	0.776

Values are presented as mean ± SD, median (IQR) or proportion (%)

^a^Two-sided *p* values for overall differences between HGI groups from ANOVA, Kruskal–Wallis or *χ*^2^ tests

There were some significant differences in baseline characteristics between the HGI groups (Table 1). Individuals in the high HGI group had the longest duration of diabetes and a higher proportion used glucose-lowering medication. More of those in the high HGI group were smokers, but fewer consumed alcohol. The prevalence of previous microvascular events was highest in the high HGI group. Almost half of the individuals in the high HGI group were of Asian origin, in contrast to a third in the low and intermediate group. HbA_1c_ levels increased from low to high HGI group and FPG was lowest in the intermediate HGI group.

### Treatment-modifying effect of HGI

Table 2 shows the adjusted HRs based on HGI group and treatment allocation (intensive or standard glucose-lowering therapy). The effect of treatment allocation on macro- and microvascular complications was similar across the three HGI groups. However, the effect of intensive therapy on mortality risk differed between HGI groups (*p* for interaction = 0.011). In the high HGI group the mortality risk was significantly lower with intensive therapy (adjusted HR 0.74 [95% CI 0.61, 0.91]; *p* = 0.003), whereas intensive treatment did not diminish risk for mortality in the low and intermediate groups. This effect remained after additional adjustment for baseline HbA_1c_ level (electronic supplementary material (ESM) Table 1). With regard to severe hypoglycaemia, absolute rates increased progressively as HGI rose, regardless of whether individuals received intensive or standard treatment. The effect of intensive treatment on the risk for severe hypoglycaemia was not different between HGI thirds, as indicated by the non-significant *p* value for interaction (0.228). To compare the predictive value of HGI with HbA_1c_, we assessed the interaction between treatment and HbA_1c_ groups using the same multivariable model (ESM Table 2). Here, there was no difference in effect of intensive treatment on mortality risk across the three HbA_1c_ groups (*p* for interaction = 0.530). Individuals with intermediate and high HbA_1c_ were at greater risk for severe hypoglycaemia when intensively treated, contrary to individuals with low HGI.

**Table 2:** Multivariable Cox proportional hazard regression analysis for major macrovascular events, major microvascular events, total mortality and severe hypoglycaemia predicted by treatment and HGI group

	Intensive treatment			Standard treatment			Adjusted HR^e^		*p* value for interaction^f^
Events by HGI group	At risk (*n*)	Events (*n*)	%^d^	At risk (*n*)	Events (*n*)	%^d^	Estimate (95% CI)	*p* value	
Major macrovascular events^a^									
Overall	5542	555	5.0	5541	587	5.3	0.95 (0.84, 1.07)	0.403	0.124
Low HGI	1873	180	4.9	1821	164	4.4	1.12 (0.91, 1.39)	0.297	
Intermediate HGI	1829	167	4.5	1867	178	4.8	0.92 (0.74, 1.14)	0.436	
High HGI	1840	208	5.6	1853	245	6.6	0.84 (0.69, 1.01)	0.059	
Major microvascular events^b^									
Overall	5542	526	4.7	5541	603	5.4	0.87 (0.77, 0.99)	0.029	0.845
Low HGI	1873	141	3.8	1821	159	4.3	0.84 (0.67, 1.06)	0.136	
Intermediate HGI	1829	148	4.0	1867	178	4.8	0.87 (0.70, 1.08)	0.216	
High HGI	1840	237	6.4	1853	266	7.2	0.91 (0.77, 1.09)	0.314	
Total mortality									
Overall	5542	494	4.5	5541	531	4.8	0.95 (0.84, 1.08)	0.420	0.011
Low HGI	1873	160	4.3	1821	137	3.7	1.17 (0.93, 1.47)	0.181	
Intermediate HGI	1829	160	4.3	1867	162	4.4	0.98 (0.79, 1.23)	0.887	
High HGI	1840	174	4.7	1853	232	6.3	0.74 (0.61, 0.91)	0.003	
Severe hypoglycaemia^c^									
Overall	5542	149	1.3	5541	81	0.7	1.82 (1.38, 2.40)	<0.0001	0.228
Low HGI	1873	34	0.9	1821	24	0.6	1.34 (0.79, 2.27)	0.276	
Intermediate HGI	1829	49	1.3	1867	28	0.8	1.82 (1.14, 2.90)	0.012	
High HGI	1840	66	1.8	1853	29	0.8	2.45 (1.57, 3.85)	<0.0001	

^a^Major macrovascular events were defined as death from a cardiovascular cause, non-fatal myocardial infarction or stroke

^b^Major microvascular events were defined as new or worsening nephro- or retinopathy

^c^Severe hypoglycaemic episodes were defined as transient dysfunction of the central nervous system with the inability to treat oneself

^d^Percentage of events respective to the total cohort (overall, *n=*11,083; low HGI, *n*=3694; intermediate HGI, *n*=3696; high HGI, *n*=3693)

^e^Model was adjusted for age, sex, ethnic origin (Asian vs non-Asian), BMI, duration of type 2 diabetes, history of macro- and microvascular events, current drinking and smoking, use of glucose-lowering drugs, use of BP-lowering drugs, systolic BP, diastolic BP, haemoglobin, renal function (eGFR), LDL-, HDL- and total cholesterol, triacylglycerol

^f^*p* value for interaction between treatment effect and HGI group

### Effect of HGI independent of treatment allocation

Table 3 shows HRs for outcomes by HGI group irrespective of treatment allocation, with the low HGI group as reference group. In the multivariable analysis, individuals in the high HGI group had a significantly higher risk for major macrovascular events, compared with the low HGI group (HR 1.26 [95% CI 1.09, 1.46]; *p* = 0.002). Further, the risk for major microvascular events and mortality was also higher in the high HGI group compared with the low HGI group (HR 1.46 [95% CI 1.26, 1.69], *p* < 0.0001 and HR 1.36 [95% CI 1.17, 1.59], *p* < 0.0001, respectively). The risk of severe hypoglycaemia did not differ between HGI groups. The effect of HGI on these complications disappeared after additional adjustment for HbA_1c_ (ESM Table 3). Again, we assessed the association between HbA_1c_ groups and adverse outcomes using the same multivariable model (ESM Table 4). Likewise, the high HbA_1c_ group had a higher risk for macro- and microvascular complications and mortality compared with the low HbA_1c_ group. HRs exceeded those seen for HGI. HbA_1c_ was not associated with the risk of severe hypoglycaemia.

**Table 3:** Cox proportional hazard regression analysis for major macrovascular events, major microvascular events, total mortality and severe hypoglycaemia predicted by HGI group (using the low group as a reference)

				Unadjusted HR		Adjusted HR^d^	
Events by HGI group	At risk (*n*)	Events (*n*)	%	Estimate 95% CI	*p* value (vs low)	Estimate 95% CI	*p* value (vs low)
Major macrovascular events^a^							
Overall	11,083	1142	10.3				
Low HGI	3694	344	9.3				
Intermediate HGI	3696	345	9.3	1.00 (0.86, 1.16)	0.989	0.97 (0.84, 1.13)	0.738
High HGI	3693	453	12.3	1.35 (1.17, 1.55)	<0.0001	1.26 (1.09, 1.46)	0.002
Major microvascular events^b^							
Overall	11,083	1129	10.2				
Low HGI	3694	300	8.1				
Intermediate HGI	3696	326	8.8	1.09 (0.93, 1.27)	0.286	1.09 (0.93, 1.27)	0.306
High HGI	3693	503	13.6	1.77 (1.53, 2.04)	<0.0001	1.46 (1.26, 1.69)	<0.0001
Total mortality							
Overall	11,083	1025	9.2				
Low HGI	3694	297	8.0				
Intermediate HGI	3696	322	8.7	1.09 (0.93, 1.27)	0.300	1.01 (0.86, 1.19)	0.865
High HGI	3693	406	11.0	1.39 (1.19, 1.61)	<0.0001	1.36 (1.17, 1.59)	<0.0001
Severe hypoglycaemia^c^							
Overall	11,083	230	2.1				
Low HGI	3694	58	1.6				
Intermediate HGI	3696	77	2.1	1.30 (0.91, 1.84)	0.144	1.25 (0.88, 1.77)	0.219
High HGI	3693	95	2.6	1.56 (1.11, 2.20)	0.010	1.33 (0.94, 1.89)	0.110

^a^Major macrovascular events were defined as death from a cardiovascular cause, non-fatal myocardial infarction or stroke

^b^Major microvascular events were defined as new or worsening nephro- or retinopathy

^c^Severe hypoglycaemic episodes were defined as transient dysfunction of the central nervous system with the inability to treat oneself

^d^Model was adjusted for age, sex, ethnic origin (Asian vs non-Asian), BMI, duration of type 2 diabetes, history of macro- and microvascular events, current drinking and smoking, use of glucose-lowering drugs, use of BP-lowering drugs, systolic BP, diastolic BP, haemoglobin, renal function (eGFR), LDL-, HDL- and total cholesterol, triacylglycerol

**Fig. 2:**
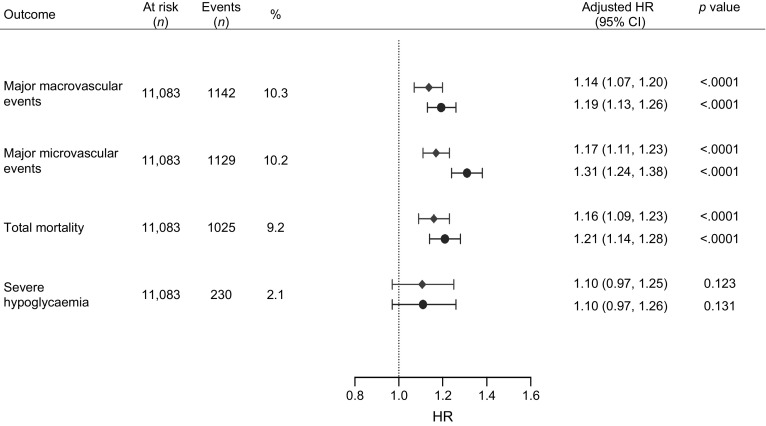
Multivariable Cox proportional hazard regression analysis for major macrovascular events, major microvascular events, total mortality and severe hypoglycaemia predicted by continuous HGI and HbA_1c_ per SD increase (1 SD of HGI is 1.20. 1 SD of HbA_1c_ is 1.56%). Major macrovascular events were defined as death from a cardiovascular cause, non-fatal myocardial infarction or stroke. Major microvascular events were defined as new or worsening nephro- or retinopathy. Severe hypoglycaemic episodes were defined as transient dysfunction of the central nervous system with the inability to treat oneself. Model was adjusted for age, sex, ethnic origin (Asian vs non-Asian), BMI, duration of type 2 diabetes, history of macro- and microvascular events, current drinking and smoking, use of glucose-lowering drugs, use of BP-lowering drugs, systolic BP, diastolic BP, haemoglobin, renal function (eGFR), LDL-, HDL- and total cholesterol, triacylglycerol. Diamonds, HGI; circles, HbA_1c_

When we considered HGI as a continuous variable, every 1 SD (=1.20 HGI) increase resulted in a 14% risk increase for microvascular complications, a 17% risk increase for macrovascular complications and a 16% risk increase for mortality (*p* < 0.0001) (Fig. 2). Continuous HGI was not associated with severe hypoglycaemia (HR 1.10 [95% CI 0.97, 1.25]; *p* = 0.123). Continuous HbA_1c_ (1 SD = 1.56% HbA_1c_) was a stronger predictor for the risk of microvascular events (HR 1.19 [95% CI 1.13, 1.26]; *p* < 0.0001), macrovascular events (HR 1.31 [95% CI 1.24, 1.38]; *p* < 0.0001) and mortality (HR 1.14 [95% CI 1.14, 1.28]; *p* < 0.0001) than HGI.

## Discussion

With this analysis of the ADVANCE trial we showed that HGI predicts diabetes-related complications, but no better than HbA_1c._ Irrespective of treatment allocation, the high HGI group (i.e. individuals with higher HbA_1c_ levels than would be expected for their given fasting glucose levels) was at higher risk for macro- and microvascular complications and mortality compared with the low HGI group. Every SD increase in HGI gave a significant 14–17% risk increase for these three outcomes. Hypothetically, this could be explained by a higher propensity for glycation of membrane proteins and lipids other than haemoglobin, with these glycation products leading to microvascular complications and atherogenesis. This effect disappeared after additional adjustment for HbA_1c_, which is in line with results on the effect of HGI in individuals with type 1 diabetes in the DCCT [22]. However, this might be considered over-adjustment, as HGI is so strongly related to HbA_1c_ (HGI is HbA_1c_ corrected for FPG). Therefore, we separately assessed the effect of HbA_1c_ on outcomes using the same model and found that HbA_1c_ was just as strong or even stronger for predicting complications in this cohort of individuals with type 2 diabetes. To our knowledge, this is the first time the predictive value of HGI has been compared with that of HbA_1c_ in this way. As HbA_1c_ does not need a population regression equation as does HGI, this makes HbA_1c_ more straightforward and convenient to use.

In our study, intensive treatment carried a lower risk for mortality in individuals with a high HGI, whereas it had no effect on mortality in individuals with a low or intermediate HGI. Thus, a high HGI identified a group of people who benefitted most from intensive HbA_1c_-lowering treatment in terms of mortality. This finding remained after additional correction for baseline HbA_1c_, but was not replicated by using HbA_1c_ as the predicting variable, as we found no interaction between treatment and HbA_1c_ groups. Overall, the estimates for the effect of intensive treatment as shown in Table 2 remained unchanged after additional adjustment for HbA_1c_ (ESM Table 1), which is not surprising given that HGI is just a linear function of HbA_1c_ and FPG (i.e. it is HbA_1c_ – (a + b × FPG), where a and b are regression coefficients). The above directly opposes the results of an analysis of the ACCORD trial, where intensive treatment was associated with a significantly higher, instead of lower, risk for mortality in participants with a high HGI [16]. Thus, the hypothesis put forward that a high HGI results in more complications due to more intensive treatment to lower HbA_1c_ than is necessary to lower plasma glucose is not supported by our study.

The inconsistency between the effect of HGI on outcomes in these two large outcome studies might be explained by important differences between ACCORD and ADVANCE. First, glucose-treatment strategies were different, although both took HbA_1c_ as predominant measure of glycaemia and both took glucose into account. Glycaemic treatment in ACCORD was intensified when HbA_1c_ level was ≥42 mmol/mol (≥6%) or when >50% of the self-monitored pre- or 2 h post-meal capillary glucose values were above a certain threshold [26].The treatment algorithm of the ADVANCE trial took discrepancies between HbA_1c_ levels and blood glucose values into account simultaneously [23]. When HbA_1c_ level was >47 mmol/mol (>6.5%) but fasting glucose was relatively low, mealtime interventions were optimised and the reliability of the tests was checked. Also ACCORD had participants who started with a higher HbA_1c_ and had a lower target HbA_1c_ in the intensive group. Further, 30% more individuals under intensive treatment received insulin in ACCORD compared with ADVANCE [5]. This is in agreement with the observation that FPG was treated more aggressively in ACCORD, with a decrease of 3.3 mmol/l from baseline to end of trial, compared with 1.9 mmol/l in ADVANCE. Moreover, in ADVANCE all participants received a sulfonylurea derivative at the start, while in ACCORD thiazolidinedione treatment was frequently used. The additional treatments differed between studies (i.e. use of aspirin and statins was substantially higher in ACCORD). Second, ACCORD was terminated prematurely, limiting the follow-up, and potentially misrepresenting estimates (no adjustment to standard errors was made for early stopping). Third, ADVANCE and ACCORD were discordant in the major findings, including mortality. In ACCORD, mortality rates were significantly higher in the intervention arm compared with the control arm [14], whereas in ADVANCE there were no significant differences in mortality between arms [23]. Post hoc, it was shown that intensively treated ACCORD participants with a high average on-treatment HbA_1c_ (>53 mmol/mol [>7%]) were at greater risk for mortality than intensively treated participants with average HbA_1c_ <53 mmol/mol (<7%) or standard-treated individuals with average HbA_1c_ >53 mmol/mol (>7%) [27]. The number of individuals experiencing severe hypoglycaemia was significantly higher with intensive treatment in both studies, but the event rates per person-year were higher in the ACCORD trial (3.5% per year with intensive treatment vs 1.0% per year in the control arm), whereas in ADVANCE rates were 0.7% per year in intensive treatment vs 0.4% per year in the control arm. We found no observable difference in the effect of HGI on severe hypoglycaemia due to intensive treatment, although the absolute rates and adjusted hazard ratios increased as HGI rose. With regard to HbA_1c_, individuals with intermediate and high HbA_1c_ were at greater risk for severe hypoglycaemia when intensively treated compared with individuals with low HbA_1c_, a finding consistent with previous literature [28].

Baseline characteristics of individuals with high HGI accorded with previous studies [16, 29]. The ethnic differences (i.e. more Asians in the high HGI group) are consistent with the observation that ethnicity influences the haemoglobin glycation, with, in general, relative higher HbA_1c_ levels in non-whites [17, 30–33]. However, regression equations in Asian (HbA_1c_ = 4.6 + 0.373 × FPG, *r*^2^ = 0.41) and non-Asian (HbA_1c_ = 4.5 + 0.340 × FPG, *r*^2^ = 0.40) participants were very similar. The combination of a slightly younger age, longer duration of diabetes (on average 2 years), more use of glucose-lowering medication and higher rates of microvascular complications suggests that individuals with high HGI might have a form of diabetes that is more difficult to treat. This in itself can be the cause of diabetes-related complications, but there is also potential for confounding, as these characteristics could well be explained by the higher HbA_1c_ levels in individuals with high HGI [22]. The HGI concept is based on the proposed inter-individual variation in haemoglobin glycation, while an adequate method for measuring glycation rate is lacking. Erythrocyte lifespan is a major determinant of the variation in haemoglobin glycation and subtle natural variation in senescence of erythrocytes is complex to quantify [34, 35]. To our knowledge, there are no studies focusing on the pathophysiological mechanism explaining both the biological variation in haemoglobin glycation as well as the reason for the possible increased risk for complications associated with higher glycation rates. This study was limited by a single FPG measurement to determine the relationship with HbA_1c_, so we could not take diurnal changes in plasma glucose into account. A measure of average glucose would have been preferred, but was not available in our data and might not be in clinical practice where individuals often use oral glucose-lowering medication only. The DCCT used seven-point glucose profiles to assess HGI [21], while ACCORD used FPG only [16].

In conclusion, we found that HGI predicted macro- and microvascular complications and mortality, but was no better than HbA_1c_, which was a stronger predictor for these outcomes. Moreover, HbA_1c_ is simpler than HGI. High HGI does predict risk for mortality with intensive treatment, but results are the opposite of those from ACCORD. Bringing all this together, the evidence does not support the clinical relevance and usefulness of HGI above HbA_1c_.

## Electronic supplementary material

ESM Tables (PDF 199 kb)

## Data Availability

The datasets analysed are available upon reasonable request and with permission of the ADVANCE Collaborative Group.

## Abbreviations

ACCORD: Action to Control Cardiovascular Risk in Diabetes
ADVANCE: Action in Diabetes and Vascular Disease: Preterax and Diamicron Modified Release Controlled Evaluation
FPG: Fasting plasma glucose
HGI: Haemoglobin glycation index
