# Supplementary material for: Haemoglobin glycation index and risk for diabetes-related complications in the Action in Diabetes and Vascular Disease: Preterax and Diamicron Modified Release Controlled Evaluation (ADVANCE) trial
Source: Diabetologia. 2018 Jan 8;61(4):780–9. doi: 10.1007/s00125-017-4539-1 (PMC6448976; doi:10.1007/s00125-017-4539-1)
Supplement: Supplementary file 1 — (PDF 199 kb) [file 125_2017_4539_MOESM1_ESM.pdf]

ESM Table 1 Multivariable Cox proportional hazard regression analysis for major macrovascular events, major microvascular events, total mortality and severe hypoglycaemia predicted by treatment and HGI group, after additional adjustment for HbA_{1c}

	Intensive treatment			Standard treatment			Adjusted HR[e]		*p* value for interaction[f]
Events by HGI group	At risk (*n*)	Events (*n*)	%[d]	At risk (*n*)	Events (*n*)	%	Estimate (95% CI)	*p* value	
Major macro-vascular events[a]									
Overall	5542	555	5.0	5541	587	5.3	0.95 (0.84, 1.07)	0.407	0.114
Low HGI	1873	180	4.9	1821	164	4.4	1.12 (0.91, 1.39)	0.285	
Intermediate HGI	1829	167	4.5	1867	178	4.8	0.92 (0.74, 1.14)	0.441	
High HGI	1840	208	5.6	1853	245	6.6	0.83 (0.69, 1.00)	0.055	
Major microvascular events[b]									
Overall	5542	526	4.7	5541	603	5.4	0.87 (0.77, 0.99)	0.030	0.825
Low HGI	1873	141	3.8	1821	159	4.3	0.84 (0.67, 1.05)	0.128	
Intermediate HGI	1829	148	4.0	1867	178	4.8	0.87 (0.70, 1.09)	0.222	
High HGI	1840	237	6.4	1853	266	7.2	0.92 (0.77, 1.09)	0.328	
Total mortality									
Overall	5542	494	4.5	5541	531	4.8	0.95 (0.84, 1.08)	0.426	0.011
Low HGI	1873	160	4.3	1821	137	3.7	1.17 (0.93, 1.48)	0.177	
Intermediate HGI	1829	160	4.3	1867	162	4.4	0.98 (0.79, 1.23)	0.892	
High HGI	1840	174	4.7	1853	232	6.3	0.74 (0.61, 0.91)	0.003	
Severe hypoglycaemia[c]									
Overall	5542	149	1.3	5541	81	0.7	1.82 (1.38, 2.40)	<.0001	0.228
Low HGI	1873	34	0.9	1821	24	0.6	1.34 (0.79, 2.27)	0.275	
Intermediate HGI	1829	49	1.3	1867	28	0.8	1.82 (1.17, 2.90)	0.012	
High HGI	1840	66	1.8	1853	29	0.8	2.45 (1.57, 3.85)	<.0001	

[a]Major macrovascular events were defined as death from a cardiovascular cause, non-fatal myocardial infarction or stroke
[b]Major microvascular events were defined as new or worsening nephro- or retinopathy
[c]Severe hypoglycaemic episodes were defined as transient dysfunction of the central nervous system with the inability to treat oneself
[d]Percentage individuals with an event respective to the total cohort of 11,083 individuals
[e]Model was adjusted for age, sex, ethnic origin (Asian vs non-Asian), BMI, duration of type 2 diabetes, history of macro- and microvascular events, current drinking and smoking, use of glucose-lowering drugs, use of BP-lowering drugs, systolic BP, diastolic BP, haemoglobin, renal function (eGFR), LDL-, HDL- and total cholesterol, triacylglycerol, HbA_{1c}
[f]*p* value for interaction between treatment effect and HGI group

ESM Table 2 Multivariable Cox proportional hazard regression analysis for major macrovascular events, major microvascular events, total mortality and severe hypoglycaemia predicted by treatment and HbA_{1c} group

	Intensive treatment			Standard treatment			Adjusted HR[e]		*p* value for interaction[f]
Events by HGI group	At risk (*n*)	Events (*n*)	%[d]	At risk (*n*)	Events (*n*)	%	Estimate (95% CI)	*p* value	
Major macro-vascular events[a]									
Overall	5542	555	5.0	5541	587	5.3	0.94 (0.84, 1.06)	0.326	0.915
Low HGI	1926	168	4.4	1891	175	4.6	0.97 (0.79, 1.20)	0.808	
Intermediate HGI	1810	167	4.6	1834	171	4.7	0.94 (0.75, 1.16)	0.558	
High HGI	1806	220	6.1	1816	241	6.7	0.92 (0.76, 1.10)	0.357	
Major microvascular events[b]									
Overall	5542	526	4.7	5541	603	5.4	0.88 (0.78, 1.00)	0.048	0.566
Low HGI	1926	132	3.5	1891	157	4.1	0.81 (0.64, 1.02)	0.075	
Intermediate HGI	1810	140	3.8	1834	144	4.0	0.97 (0.77, 1.23)	0.788	
High HGI	1806	254	7.0	1816	302	8.3	0.88 (0.74, 1.04)	0.129	
Total mortality									
Overall	5542	494	4.5	5541	531	4.8	0.93 (0.82, 1.06)	0.277	0.530
Low HGI	1926	147	3.9	1891	143	3.7	1.04 (0.82, 1.31)	0.761	
Intermediate HGI	1810	158	4.3	1834	173	4.7	0.88 (0.71, 1.10)	0.261	
High HGI	1806	189	5.2	1816	215	5.9	0.89 (0.73, 1.08)	0.240	
Severe hypoglycaemia[c]									
Overall	5542	149	1.3	5541	81	0.7	1.85 (1.40, 2.45)	<.0001	0.018
Low HGI	1926	34	0.9	1891	32	0.8	1.08 (0.66, 1.76)	0.758	
Intermediate HGI	1810	55	1.5	1834	27	0.7	2.02 (1.27, 3.20)	0.003	
High HGI	1806	60	1.7	1816	22	0.6	2.92 (1.77, 4.80)	<.0001	

[a]Major macrovascular events were defined as death from a cardiovascular cause, non-fatal myocardial infarction or stroke
[b]Major microvascular events were defined as new or worsening nephro- or retinopathy
[c]Severe hypoglycaemic episodes were defined as transient dysfunction of the central nervous system with the inability to treat oneself
[d]Percentage individuals with an event respective to the total cohort of 11,083 individuals
[e]Model was adjusted for age, sex, ethnic origin (Asian vs non-Asian), BMI, duration of type 2 diabetes, history of macro- and microvascular events, current drinking and smoking, use of glucose-lowering drugs, use of BP-lowering drugs, systolic BP, diastolic BP, haemoglobin, renal function (eGFR), LDL-, HDL- and total cholesterol, triacylglycerol
[f]*p* value for interaction between treatment effect and HGI group

ESM Table 3 Cox proportional hazard regression analysis for major macrovascular events, major microvascular events, total mortality and severe hypoglycaemia predicted by HGI group (using the low group as a reference), after additional adjustment for HbA_{1c}

				Unadjusted HR		Adjusted HR[d]	
Events by HGI group	At risk (*n*)	Events (*n*)	%	Estimate 95% CI	*p* value (vs low)	Estimate 95% CI	*p* value (vs low)
Major macro-vascular events[a]							
Overall	11083	1142	10.3				
Low HGI	3694	344	9.3				
Intermediate HGI	3696	345	9.3	1.00 (0.86, 1.16)	0.989	0.89 (0.77, 1.04)	0.160
High HGI	3693	453	12.3	1.35 (1.17, 1.55)	<0.0001	0.98 (0.81, 1.17)	0.797
Major micro-vascular events[b]							
Overall	11083	1129	10.2				
Low HGI	3694	300	8.1				
Intermediate HGI	3696	326	8.8	1.09 (0.93, 1.27)	0.286	0.95 (0.81, 1.12)	0.531
High HGI	3693	503	13.6	1.77 (1.53, 2.04)	<0.0001	0.93 (0.78, 1.12)	0.458
Total mortality							
Overall	11083	1025	9.2				
Low HGI	3694	297	8.0				
Intermediate HGI	3696	322	8.7	1.09 (0.93, 1.27)	0.300	0.94 (0.79, 1.10)	0.435
High HGI	3693	406	11.0	1.39 (1.19, 1.61)	<0.0001	1.07 (0.88, 1.30)	0.489
Severe hypoglycaemia[c]							
Overall	11083	230	2.1				
Low HGI	3694	58	1.6				
Intermediate HGI	3696	77	2.1	1.30 (0.91, 1.84)	0.144	1.23 (0.86, 1.77)	0.256
High HGI	3693	95	2.6	1.56 (1.11, 2.20)	0.010	1.29 (0.84, 1.97)	0.247

[a]Major macrovascular events are defined as death from a cardiovascular cause, non-fatal myocardial infarction or stroke
[b]Major microvascular events are defined as new or worsening nephro- or retinopathy
[c]Severe hypoglycaemic episodes were defined as transient dysfunction of the central nervous system with the inability to treat oneself
[d]Model was adjusted for age, sex, ethnic origin (Asian vs non-Asian), BMI, duration of type 2 diabetes, history of macro- and microvascular events, current drinking and smoking, use of glucose-lowering drugs, use of BP-lowering drugs, systolic BP, diastolic BP, haemoglobin, renal function (eGFR), LDL-, HDL- and total cholesterol, triacylglycerol, HbA_{1c}

ESM Table 4 Cox proportional hazard regression analysis for major macrovascular events, major microvascular events, total mortality and severe hypoglycaemia predicted by HbA_{1c} group (using the low group as a reference)

				Unadjusted HR		Adjusted HR[d]	
Events by HGI group	At risk (*n*)	Events (*n*)	%	Estimate 95% CI	*p* value (vs low)	Estimate 95% CI	*p* value (vs low)
Major macro-vascular events[a]							
Overall	11083	1142	10.3				
Low HGI	3817	343	9.0				
Intermediate HGI	3644	338	9.3	1.03 (0.89, 1.20)	0.696	0.96 (0.83, 1.12)	0.620
High HGI	3622	461	12.7	1.45 (1.26, 1.67)	<.0001	1.38 (1.20, 1.60)	<.0001
Major micro-vascular events[b]							
Overall	11083	1129	10.2				
Low HGI	3817	289	7.6				
Intermediate HGI	3644	284	7.8	1.03 (0.88, 1.22)	0.710	0.96 (0.82, 1.14)	0.671
High HGI	3622	556	15.4	2.17 (1.88, 2.50)	<.0001	1.79 (1.54, 2.08)	<.0001
Total mortality							
Overall	11083	1025	9.2				
Low HGI	3817	290	7.6				
Intermediate HGI	3644	331	9.1	1.20 (1.02, 1.40)	0.024	1.12 (0.96, 1.32)	0.151
High HGI	3622	404	11.2	1.50 (1.29, 1.74)	<.0001	1.50 (1.28, 1.75)	<.0001
Severe hypoglycaemia[c]							
Overall	11083	230	2.1				
Low HGI	3817	66	1.7				
Intermediate HGI	3644	82	2.3	1.23 (0.88, 1.71)	0.229	1.14 (0.81, 1.60)	0.450
High HGI	3622	82	2.3	1.18 (0.84, 1.67)	0.333	1.07 (0.75, 1.53)	0.724

[a]Major macrovascular events are defined as death from a cardiovascular cause, non-fatal myocardial infarction or stroke
[b]Major microvascular events are defined as new or worsening nephro- or retinopathy
[c]Severe hypoglycaemic episodes were defined as transient dysfunction of the central nervous system with the inability to treat oneself
[d]Model was adjusted for age, sex, ethnic origin (Asian vs non-Asian), BMI, duration of type 2 diabetes, history of macro- and microvascular events, current drinking and smoking, use of glucose-lowering drugs, use of BP-lowering drugs, systolic BP, diastolic BP, haemoglobin, renal function (eGFR), LDL-, HDL- and total cholesterol, triacylglycerol
